# Supplementary material for: Cryo-electron tomography reveals coupled flavivirus replication, budding and maturation
Source: bioRxiv. 2024 Oct 13:2024.10.13.618056. Preprint. [Version 1] doi: 10.1101/2024.10.13.618056 (PMC11482891; doi:10.1101/2024.10.13.618056)
Supplement: Supplementary file 1 [file NIHPP2024.10.13.618056V1-supplement-1.pdf]

bioRxiv preprint doi: https://doi.org/10.1101/2024.10.13.618056; this version posted October 13, 2024. The copyright holder for this preprint (which was not certified by peer review) is the author/funder, who has granted bioRxiv a license to display the preprint in perpetuity. It is made available under aCC-BY 4.0 International license.

Supplementary figures and movies

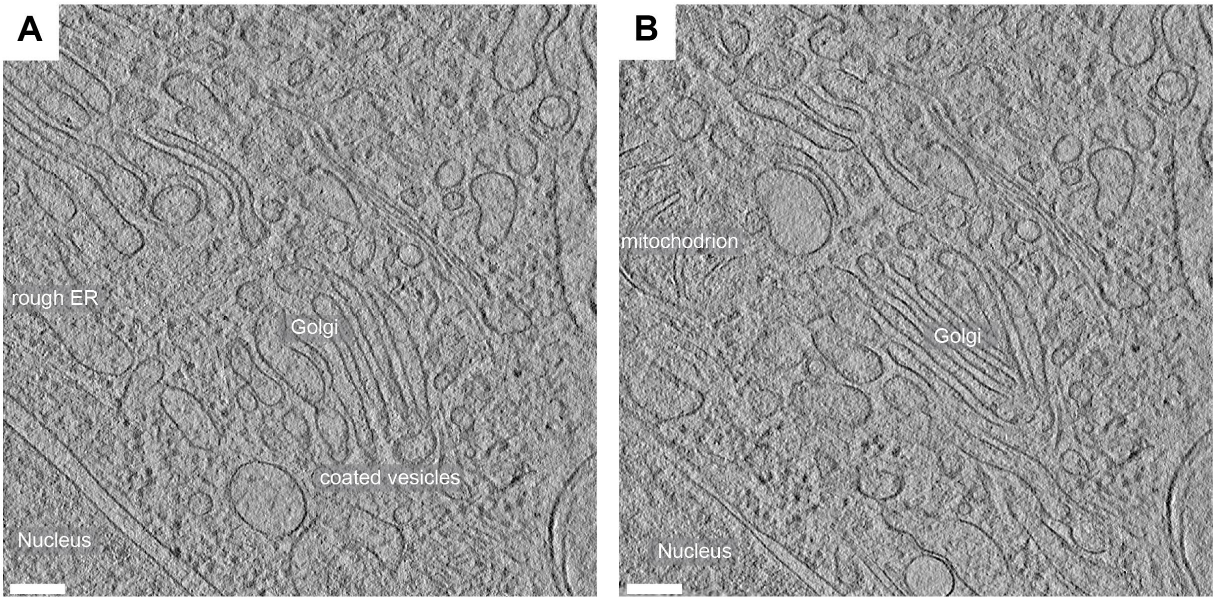

Figure S1: cryo-ET of uninfected A549 cells. (A-B) Slices from two tomograms of uninfected A549 cells reveal typical cytoplasmic features, as indicated, including a non-dilated ER and *bona fide* Golgi cisternae with typical morphology. Scale bars,100 nm.

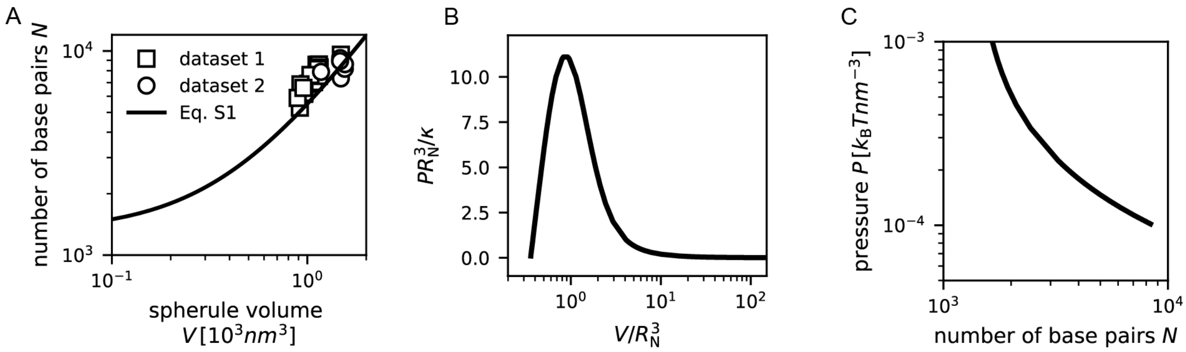

Figure S2. Pressure exerted by an RNA strand. (A) Relation between number of RNA base pairs and RO volume. The data is reproduced from Laurent *et al*[32]. We note that in Laurent *et al*[32] the RNA length is shown, while here the number of base pairs is shown, assuming an interbasepair distance of 2.56 Å. (B) Relation between the scaled pressure and the scaled volume. The details of the underlying model are presented in Laurent *et al*[32]. (C) Relation between number of RNA base pairs N and pressure P, where we use the results from (A-B) to convert the RO volume into number of RNA base pairs.

bioRxiv preprint doi: https://doi.org/10.1101/2024.10.13.618056; this version posted October 13, 2024. The copyright holder for this preprint (which was not certified by peer review) is the author/funder, who has granted bioRxiv a license to display the preprint in perpetuity. It is made available under aCC-BY 4.0 International license.

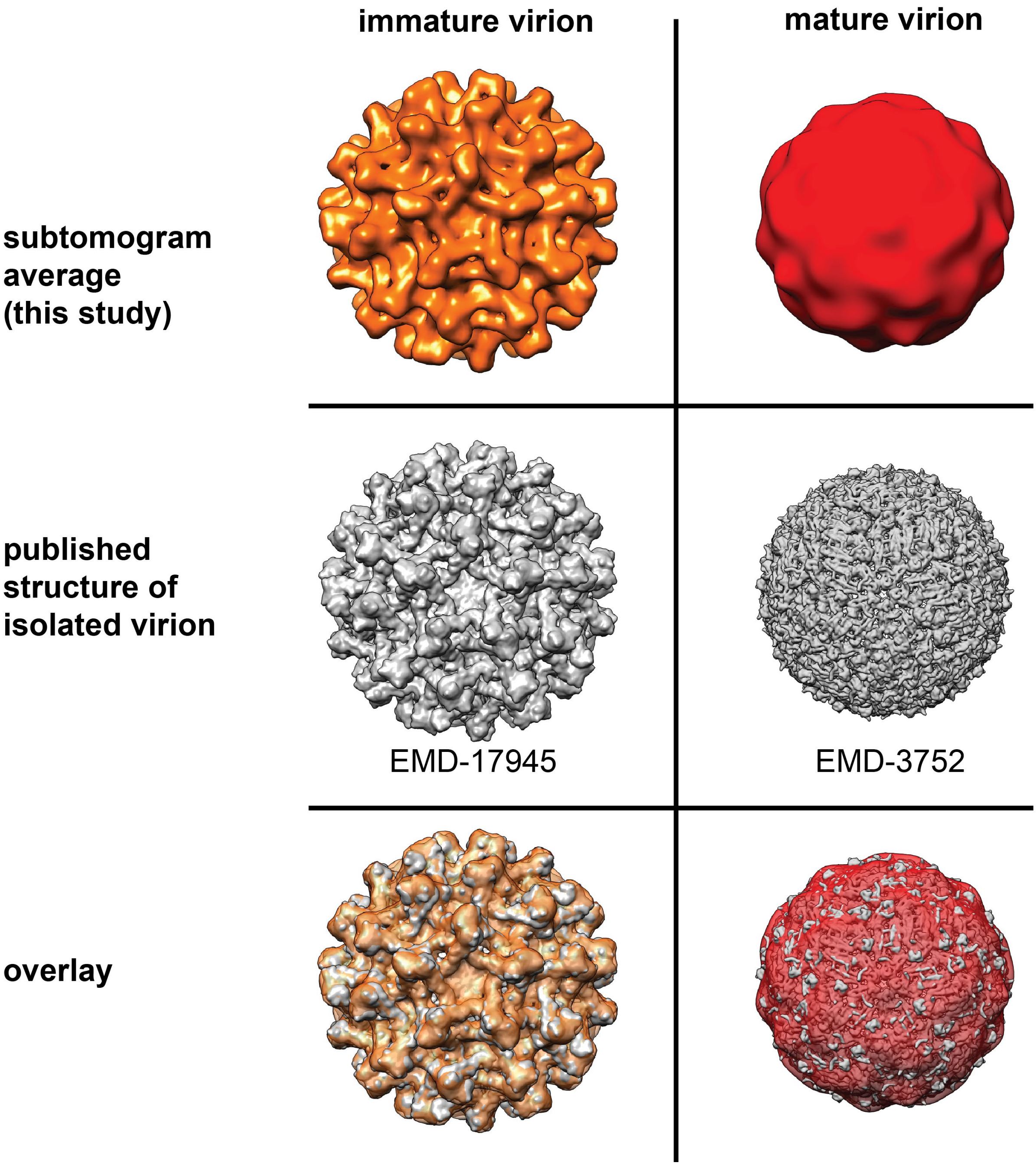

Figure S3: Comparison of cellular subtomogram averages with isolated virion structures. The cellular subtomogram averages from this study (top row) are compared to low-pass filtered published structures of immature and mature TBEV, from Fuzik *et al*[25] and Anastasina *et al*[23], respectively (mid row). The overlays (bottom row) were created using the Align to Volume command, and are shown with the subtomogram averages in semi-transparent surface representation.

bioRxiv preprint doi: https://doi.org/10.1101/2024.10.13.618056; this version posted October 13, 2024. The copyright holder for this preprint (which was not certified by peer review) is the author/funder, who has granted bioRxiv a license to display the preprint in perpetuity. It is made available under aCC-BY 4.0 International license.

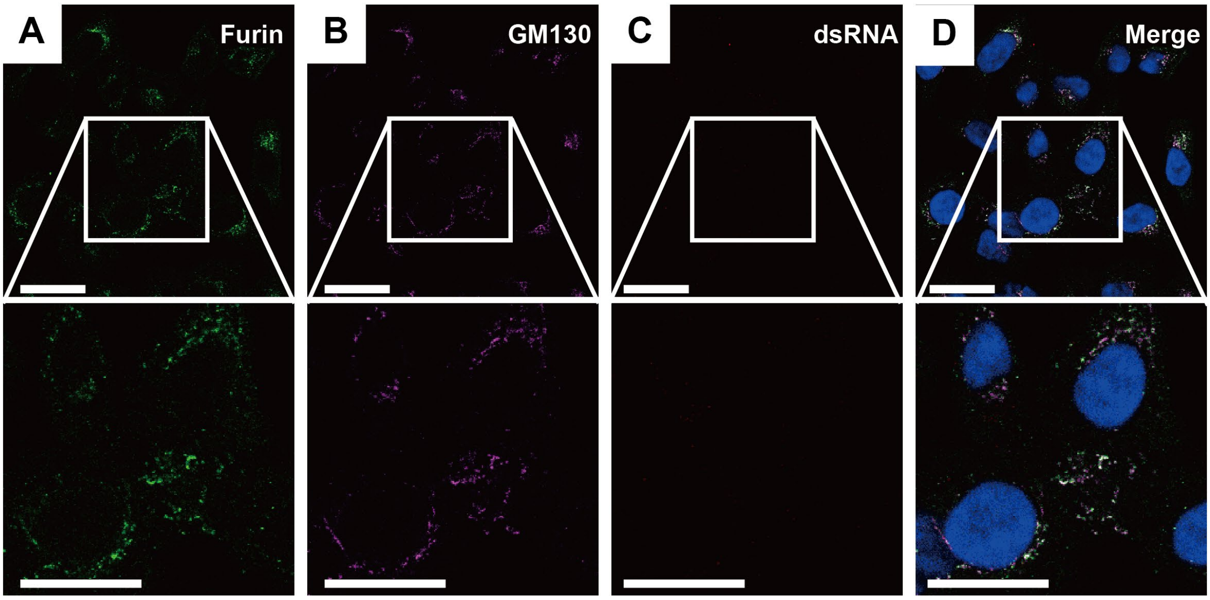

Figure S4: Furin localization in uninfected cells. Immunofluorescence microscopy of uninfected cells showing furin (A) and Golgi marker GM130 (B) and their colocalization in the absence of viral infection (D). Additional channels contain dsRNA staining (C) and DAPI staining of cell nuclei (D). Scale bars, 10 µm.

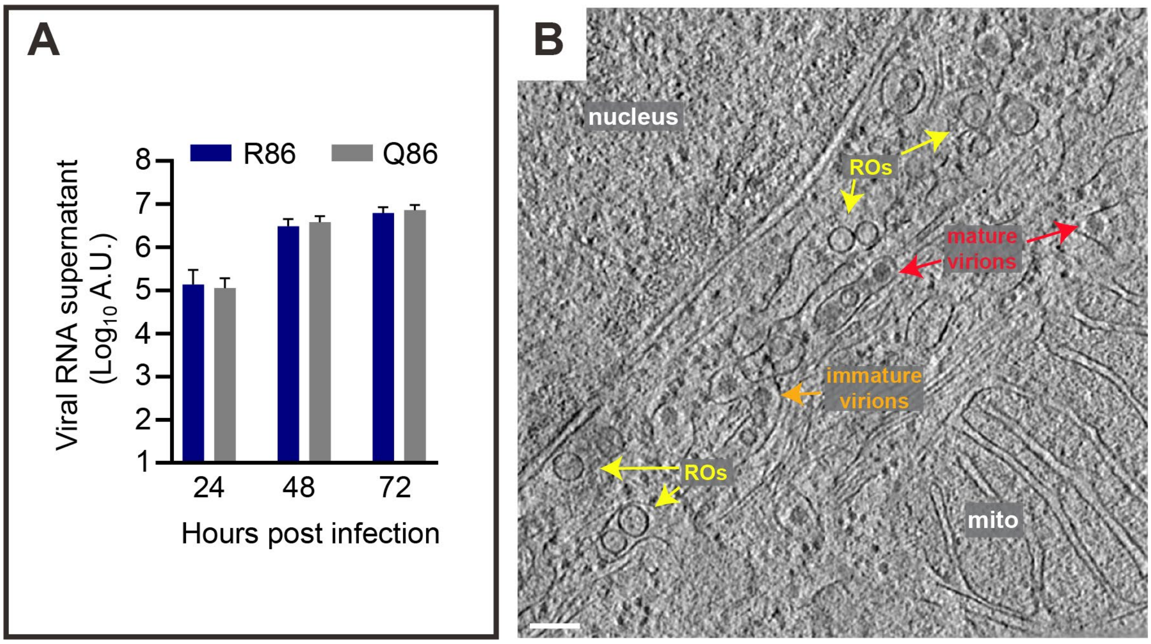

Figure S5: Data on rLGTV$^{T:prME}$ Q86 and R86. (A) Growth kinetics of rLGTV$^{T:prME}$ R86 and Q86 upon infection of A549 at MOI 1, quantitated as the amount of viral RNA in supernatant per qPCR. (B) Slice from a tomogram of a cell infected with rLGTV$^{T:prME}$ R86 at 24 h p.i. showing various cytoplasmic and virus-related features, as indicated. Scale bar 100 nm.

bioRxiv preprint doi: https://doi.org/10.1101/2024.10.13.618056; this version posted October 13, 2024. The copyright holder for this preprint (which was not certified by peer review) is the author/funder, who has granted bioRxiv a license to display the preprint in perpetuity. It is made available under aCC-BY 4.0 International license.

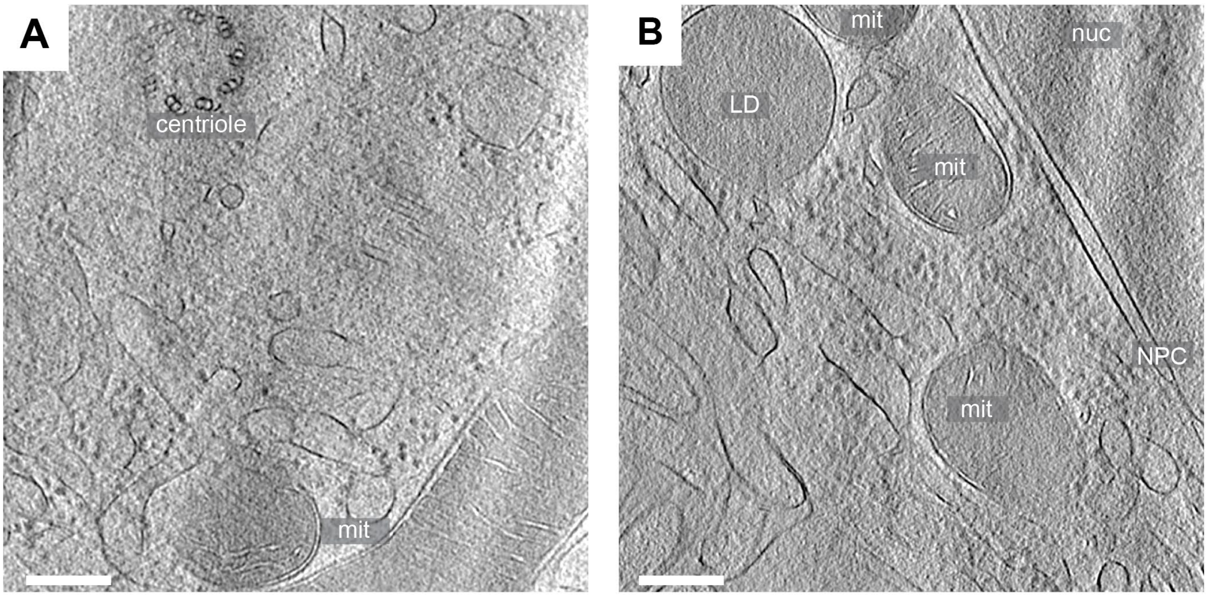

Figure S6: Features unrelated to infection in cryo-electron tomograms of *ex vivo* brain tissue. (A-B) Slices from two tomograms of high-pressure frozen choroid plexus from LGTV-infected *Ifnar*$^{-/-}$ mice. The features indicated are a centriole, several mitochondria (mit), a lipid droplet (LD), the peripheral area of a nucleus (nuc) and the nuclear envelope including one nuclear pore complex (NPC). Scale bar, 100 nm.

Movie S1. Tomographic volume corresponding to the slice shown in Fig. 1A.

Movie S2. The segmentation shown in Fig. 1D.

Movie S3. Tomographic volume corresponding to the slice shown in Fig. 3A.

Movie S4. The segmentation shown in Fig. 3B.

Movie S5. Tomographic volume corresponding to the slice shown in Fig. 4G.

Movie S6. The segmentation shown in Fig. 4H.

Movie S7. Tomographic volume corresponding to the slices shown in Fig. 5F-G.

Movie S8. The segmentation shown in Fig. 5H.

Movie S9. Tomographic volume corresponding to the slice shown in Fig.6B.
